# Transcriptomics and Metabolomics Analysis Reveal the Mechanism of Petal Number Variation in *Gardenia jasminoides*

**DOI:** 10.3390/metabo16020130

**Published:** 2026-02-13

**Authors:** Bo Gao, Yi Lu, Wenhuan Lai, Yiwen Liao, Liang Dong, Qigong Zhang, Shuangquan Zou, Xiaoxing Zou

**Affiliations:** Fujian Colleges and Universities Engineering Research Institute of Conservation and Utilization of Natural Bioresources, College of Forestry, Fujian Agriculture and Forestry University, Fuzhou 350002, China; 17568015413@163.com (B.G.); m17764041996@163.com (Y.L.); lwhcll@163.com (W.L.); lywnhm@163.com (Y.L.); 18760132106@163.com (L.D.); fafuqgz@163.com (Q.Z.); zou@fafu.edu.cn (S.Z.)

**Keywords:** gardenia flower, petal number, transcriptome, metabolome

## Abstract

**Background/Objectives**: This study was based on the joint analysis of transcriptome and metabolome to explore the key genes and metabolic pathways of gardenia single flower petal number variation and to explore the possible mechanism of floral organ variation. **Methods**: Five, six, and seven petals of single-flower gardenia were selected as test materials for transcriptome and metabolome determination to excavate the key genes in regulating petal number in gardenia. **Results**: Metabolomic analysis identified triethylamine, succinic acid, succinylaldehyde, 2-phenylethanol, and o-xylene as the top five differentially expressed metabolites affecting petal number variation in gardenia. In the KEGG enrichment analysis, gardenia five, six, and seven DEGs were mainly enriched in amphetamine biosynthesis, the biosynthesis of plant secondary metabolites; transcriptome results showed that the identified differential transcription factors mainly come from NAC, ERF, C2H2, MYB, and MADS-box gene families; the expression of *GjMADS50*, *GjMADS59*, and *GjERF28* changed with the increase in petal number. The commonality between gardenia five, six, and seven flowers exceeded the difference, and the expression pattern of MADS-box and ERF gene family members was the upregulation of *GjERF28*, *GjERF39*, and *GjMADS67* and downregulation of *GjMADS50*, *GjMADS59*, and *GjMADS60*. **Conclusions**: We propose that ERF transcription factors may determine the initial number of petal primordia by mediating gibberellin biosynthesis or signaling, thereby coordinately regulating floral meristem activity and specific metabolic states.

## 1. Introduction

As the characteristic reproductive and ornamental organs of angiosperms, flowers develop under the orchestration of a series of conserved and complex gene regulatory networks [1,2]. Morphologically, a typical flower is composed of four distinct parts: the calyx, corolla, androecium, and gynoecium. The canonical ABC and ABCDE models have successfully elucidated the determination mechanisms of floral organ identity [3,4], laying the foundation for understanding the origin and transformation of floral organs. However, while these classical models primarily address the determination of organ identity, the regulatory mechanisms governing the natural variation in floral organ number remain largely unclear in non-model plants.

In ornamental plants, variation in petal number is a common phenotypic trait regulated by the dual interplay of intrinsic genetic programs and extrinsic environmental signals [5,6]. Double flowers represent one of the earliest recorded floral mutants, often manifesting a double-flowered phenotype through corolla duplication, stamen petaloidy, and pistil petaloidy [7,8,9,10]. As an economic plant possessing both high ornamental and significant medicinal value, *Gardenia jasminoides* Ellis exhibits diverse variations in its double-flowered forms; in contrast, the petal number in single-flowered varieties, with occasional natural variants, remains relatively stable, and the underlying molecular regulatory mechanisms remain obscure. Despite long-standing observations of this phenotypic variation, research into the molecular mechanisms governing petal number formation in single-flowered gardenia remains in its infancy, and the underlying molecular genetic basis and systematic metabolic regulatory networks have yet to be elucidated. Identifying the key regulatory genes driving variation in petal primordia number and clarifying whether this developmental process is accompanied by specific hormonal signal transitions and changes in secondary metabolite accumulation is central to understanding the developmental plasticity of gardenia flowers. Furthermore, this knowledge lays a critical theoretical foundation for the targeted improvement of ornamental traits through molecular design breeding.

Traditional single-omics analyses often possess inherent limitations when deciphering such complex traits, failing to systematically unveil the complete causal chain from gene regulation to metabolic phenotypes. Integrated multi-omics analysis provides a robust tool to address this challenge, capable of directly capturing the physiological and developmental states of cells [11]. The conjoint analysis of these datasets not only enables cross-validation at the data level but also facilitates the systematic inference of potential regulatory relationships through the construction of gene-metabolite association networks. Consequently, this approach efficiently elucidates the underlying mechanisms of trait formation and identifies core candidate regulatory factors [12].

Therefore, this study aims to systematically elucidate the molecular regulatory basis underlying natural variation in petal number. By integrating transcriptomic and metabolomic analyses, we investigated the differential profiles of metabolites and key genes among five-, six-, and seven- petaled gardenia flowers. The findings not only provide critical candidate genes for the molecular breeding of gardenia but also offer valuable insights into the mechanisms governing the developmental plasticity of floral organ number in plants.

## 2. Materials and Methods

### 2.1. Materials

**Figure 1:**
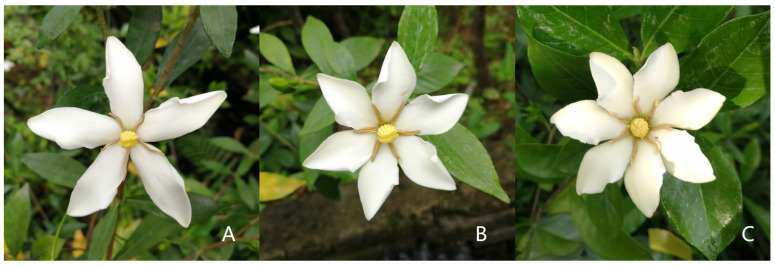
Gardenia flower of the same plant at the same period. (**A**): Five-petaled flower; (**B**): six-petaled flower; (**C**): seven-petaled flower.

Single-flowered genotypes of gardenia originating from the Quanzhou germplasm were uniformly maintained and subsequently cultivated under controlled conditions at the Fujian Agriculture and Forestry University. At the full-bloom stage of gardenia, flowers with five (Fr_5), six (Fr_6), and seven (Fr_7) petals from the same plant were collected (Figure 1). Each sample type included three biological replicates, which were immediately transferred into cryogenic tubes, flash-frozen in liquid nitrogen, and stored at −80 °C for transcriptome and metabolome sequencing.

### 2.2. Transcriptome Sequencing and Analysis Methods

The original data was downloaded using the cloud platform of Shanghai Pasenno Biotechnology Co. LTD (Shanghai, China) (https://www.genescloud.cn/ (accessed on 6 May 2023)). The sequencing data were filtered using the SOAPnuke V1.4.0 software. The reference genome was gardenia (V1, ID53980) [13], and the clean reads were aligned to the reference genome using the HISAT2 software. Open reading frames (ORFs) of the assembled unigenes were predicted, followed by functional annotation through sequence alignment against the Plant Resistance Gene database (PRGdb) using appropriate bioinformatic tools.

The Poisson distribution method was used to screen the differentially expressed genes. Multiple differential expressions among different samples were calculated based on the FPKM value (FDR ≤ 0.001, and the multiple difference was more than two times). The GO functional classification was performed on the differentially expressed genes and the enrichment analysis was conducted; the biological pathway classification and enrichment analysis were performed on the differentially expressed genes (significant enrichment: Qvalue ≤ 0.05).

### 2.3. Metabolome Sequencing and Analysis Methods

The original data download was the same as in Section 2.2. Pairwise sample distances were calculated based on the Euclidean distance metric, and hierarchical clustering was conducted to generate a dendrogram, thereby evaluating the degree of similarity among samples. Agglomerative hierarchical clustering was performed to classify metabolites across all samples and groups, and relative metabolite abundances under different experimental conditions were used to indicate the corresponding metabolic levels.

To extract the most informative features from the dataset, dimensionality reduction and classification analyses were conducted. Prior to analysis, the data were auto-scaled, and principal component analysis (PCA) was applied as the major method for multivariate statistical evaluation. In search for differential metabolites from the first-level substance list of the sample (*p* value and VIP), metabolites were determined based on MS/MS fragmentation patterns and databases such as HMDB, massbank, LipidMaps, and mzcloud. Screening was conducted based on the preset *p* value and VIP threshold in the statistical test. The metabolic pathways of the two groups of differential metabolites were analyzed by using the hypergeometric test and the MetPA database. According to the MetPA analysis results and the relative response values of metabolic pathways, the correlation coefficients between metabolic pathways were calculated.

## 3. Results

### 3.1. Evaluation of Transcriptome Sequencing Data Quality

The original data obtained by sequencing the nine samples on the machine were screened and filtered, respectively, by removing sequences with joints and low quality; 36,357,562 to 43,485,564 high-quality sequences were obtained (Table 1). The proportion of it in the original sequence was calculated to be 93.28~93.47%, and the number of high-quality sequence bases was 5,609,302,500~6,426,642,600. The base percentage (Q20) with an identification accuracy rate above 99% is ≥98%, and the base percentage (Q30) with an identification accuracy rate above 99.9% is ≥94%. The alignment rate of high-quality reads to the reference genome ranged from 92.66% to 94.01% (Table 2), while the proportion of reads mapped to multiple genomic locations accounted for 4.25% to 5.67% of the total value. Among the mapped reads, 94.33% to 95.75% were uniquely aligned to a single genomic locus, whereas 86.42% to 88.31% of the total reads were mapped to annotated gene regions, indicating a high proportion of gene-associated transcriptional reads. A total of 11.69–13.58% of reads were mapped to intergenic regions, whereas 95.57–96.58% were aligned to exonic regions. The high proportion of exonic reads suggests that the sequencing data are of high integrity and suitable for downstream analyses.

### 3.2. Analysis of Differential Gene Expression

**Figure 2:**
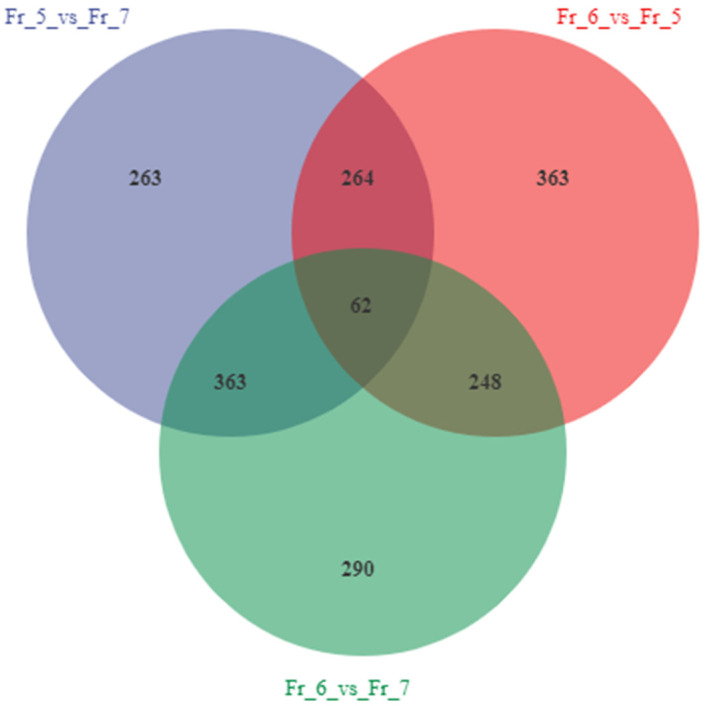
Venn diagram of sample differential genes.

DESeq was used to conduct the differential analysis of gene expression, and the number of differentially expressed genes among the three comparison groups of Fr_5 vs. Fr_7, Fr_6 vs. Fr_5, and Fr_6 vs. Fr_7 was statistically analyzed. The Venn diagram was used to represent the number of shared and unique differentially expressed genes among the three comparison groups (Figure 2). The three groups of differentially expressed genes were 952 (440 upregulated genes and 512 downregulated genes), 937 (569 upregulated genes and 368 downregulated genes), and 963 (558 upregulated genes and 405 downregulated genes), respectively. The results indicate that the six-petal gardenia has more upregulated genes than downregulated genes compared to the five-petal and seven-petal gardenia. The reduction in the number of genes regulating six-petaled gardenia is presumed to contribute to the emergence of the five-petaled and seven-petaled gardenia phenotypes.

The number of differentially expressed genes shared by all three groups is 62, while the numbers of unique differentially expressed genes in Fr_5 vs. Fr_7, Fr_6 vs. Fr_5, and Fr_6 vs. Fr_7 are 263, 363, and 290, respectively. The results indicate that the similarity between six-petaled and five-petaled gardenia flowers is higher than that between six-petaled and seven-petaled gardenia flowers.

### 3.3. Differential Metabolic Pathway Analysis of Petal Number Variation

The GO enrichment analysis was performed on the differentially expressed genes between each pair of samples, and the GO classification was conducted according to cellular component, molecular function, and biological process (Table 3). Among them, the most significant GO terms in each GO category for the differentially expressed genes between Fr_5 and Fr_7 were the cell wall, glucose binding, and response to oxygen-containing compound. The most significant GO terms in each GO category for the differentially expressed genes between Fr_6 and Fr_5 were the cell periphery, transmembrane transporter activity, and transmembrane transport. The most significant GO terms in each GO category for the differentially expressed genes between Fr_6 and Fr_7 were the cell exterior, glucosyltransferase activity, and response to jasmonic acid.

**Figure 3:**
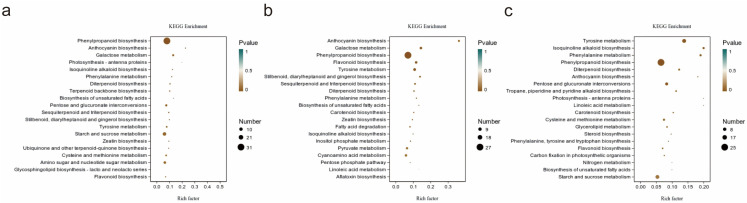
Factor diagram of differential genes. (**a**): Fr_5 vs. Fr_7; (**b**): Fr_6 vs. Fr_5; (**c**): Fr_6 vs. Fr_7.

The KEGG pathway enrichment analysis was performed on the differentially expressed genes between each pair of samples, presenting the top 20 KEGG pathways (Figure 3). The degree of enrichment was measured by the enrichment factor (Rich factor), *p*-value, and the number of genes enriched in the pathway. A larger Rich factor indicates a higher degree of enrichment; the *p* value typically ranges from 0 to 1, with values closer to 0, indicating more significant enrichment. The differentially expressed genes from the three groups were primarily enriched in phenylpropanoid biosynthesis. The secondary enriched pathways for each group were as follows: for Fr_5 vs. Fr_7, the differential genes were enriched in starch and sucrose metabolism; for Fr_6 vs. Fr_5, the differential genes were enriched in tyrosine metabolism, flavonoid biosynthesis, and galactose metabolism; and for Fr_6 vs. Fr_7, the differential genes were enriched in tyrosine metabolism, among other pathways.

### 3.4. The Analysis of Differentially Expressed Genes Underlying Petal Number Variation and the Expression Level in the Sample

Among the 47 gene families identified in the transcriptome sequencing data (Table 4), a total of 35 families encompassing 167 transcription factors were detected in the comparison between Fr_5 and Fr_7, with 80 transcription factors being upregulated and 87 being downregulated. In the comparison between Fr_6 and Fr_5, 37 families comprising 159 transcription factors were identified, including 89 upregulated and 70 downregulated factors. For the comparison between Fr_6 and Fr_7, 40 families involving 199 transcriptional genes were identified, of which 113 were upregulated and 86 were downregulated.

**Figure 4:**
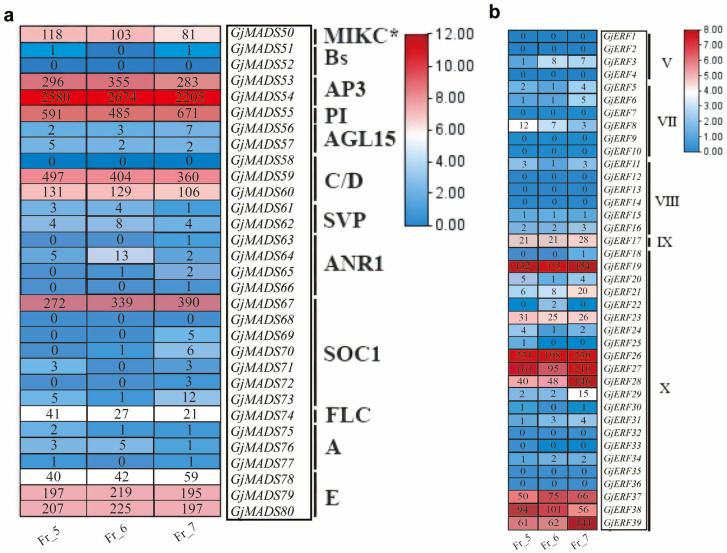
Expression levels of MADS-box and ERF family genes for Fr_5, Fr_6, and Fr_7. (**a**): MADS-box family; (**b**): ERF family.

In the fully bloomed flowers of Fr_5, Fr_6, and Fr_7, the genes with relatively high expression levels included class B genes (*GjMADS53*, *GjMADS54*, and *GjMADS55*), class C/D genes (*GjMADS59* and *GjMADS60*), the *SOC1* gene (*GjMADS67*), and class E genes (*GjMADS79* and *GjMADS80*), all of which belong to the MADS-box gene family (Figure 4a). According to the classification of differentially expressed gene families, members of the ERF subfamily belonging to the X branch—such as *GjERF19*, *GjERF26*, *GjERF27*, *GjERF28*, *GjERF37*, *GjERF38*, and *GjERF39*—also exhibited high expression (Figure 4b). As the petal number increased, the expression levels of *GjMADS50*, *GjMADS59*, and *GjMADS60* gradually decreased, whereas the expression of *GjMADS67*, *GjERF28*, and *GjERF39* increased correspondingly with the number of petals.

### 3.5. Metabolomic Data and Sample Differential Metabolite Analysis

**Figure 5:**
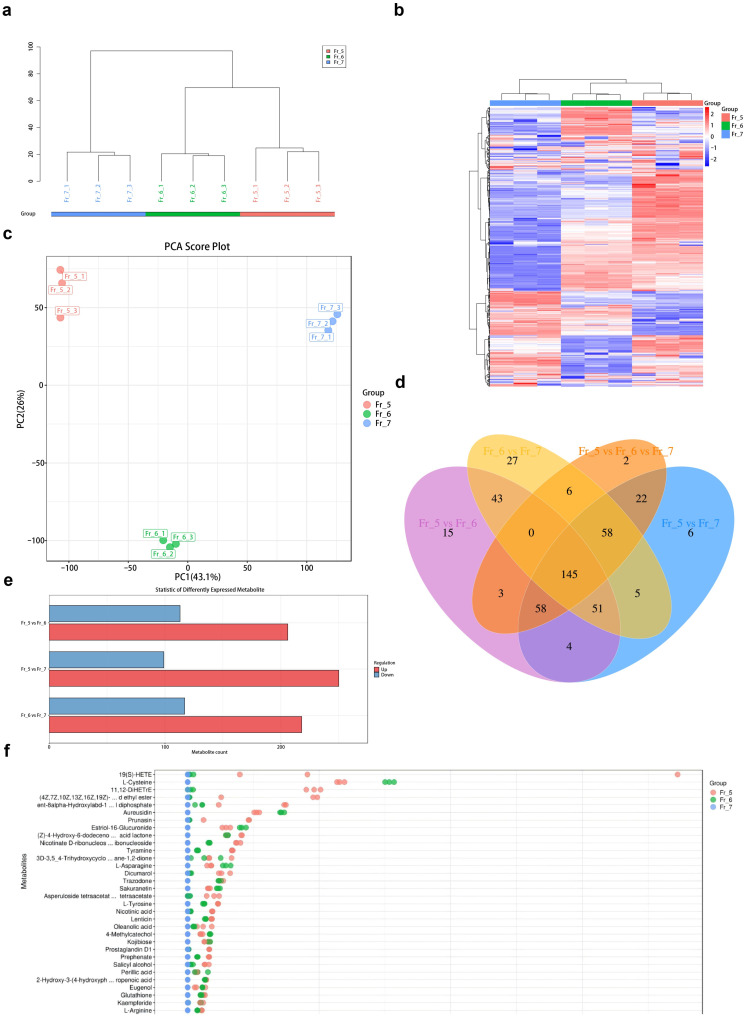
(**a**): Sample hierarchical cluster tree; (**b**): Cluster heat map of sample metabolites, red to blue indicates a gradual decrease in expression level; (**c**): PCA score diagram of Fr_5, Fr_6, and Fr_7, the horizontal axis represents the decomposition degree of the first main component, and the vertical axis represents the decomposition degree of the second main component, dots represent experimental samples, and colors represent different groups; (**d**): Venn diagram of differential metabolites; (**e**): Bar chart of statistical analysis of differential metabolites in each group, the horizontal axis shows the number of differential metabolites, and the vertical axis shows the grouping comparison conditions; (**f**): Z-score diagram, the vertical axis is the name of the metabolite, and the color of the dot represents different groups, the horizontal axis is the value of the relative content of the metabolite in the group converted by Z-score, the further to the right, the more the metabolite is in the group.

Chromatographically separated components were subjected to mass spectrometric analysis to collect data and generate mass spectra. The most intense ions at each time point in the mass spectrum were selected for continuous plotting, resulting in the base peak chromatogram (BPC). The more similar the trends of the lines in the chromatogram, the better the reproducibility of the samples, indicating reliable results. The three biological replicates of each sample were closely clustered, and the similarity between replicates was high. The similarity between the Fr_5 and Fr_6 samples was greater than that between the Fr_5 and Fr_7 samples (Figure 5a). The metabolites of the three biological replicates of each sample were relatively similar (Figure 5b); however, there were certain differences in the metabolites among the three samples. The metabolites of the Fr_5 and Fr_6 samples were more similar to each other.

In the principal component analysis (PCA) (Figure 5c), the samples within the Fr_5 and Fr_6 groups were more tightly clustered, while the samples between groups were more dispersed, indicating that the results are relatively reliable. Metabolite profiling of gardenia flowers with five, six, and seven petals was performed using MS/MS-based identification, resulting in the detection of a total of 572 metabolites. The number of differential metabolites between the four comparison groups, Fr_5 vs. Fr_7, Fr_6 vs. Fr_5, Fr_6 vs. Fr_7, and Fr_5 vs. Fr_6 vs. Fr_7 (Figure 5d), was calculated. A Venn diagram was used to represent the shared and unique differential metabolites among the four groups (Figure 5e). The total number of differential metabolites for the four groups were 349 (250 upregulated genes and 99 downregulated genes), 319 (206 upregulated genes and 113 downregulated genes), 315 (218 upregulated genes and 117 downregulated genes), and 294, respectively. The number of differential metabolites commonly shared by the four groups was 145, which corresponds to the number of main metabolites of gardenia. The number of unique differential metabolites for Fr_5 vs. Fr_7, Fr_6 vs. Fr_5, and Fr_6 vs. Fr_7 were 204, 174, and 170, respectively. The number of differential metabolites shared by the four groups is 145, which represents the main metabolites of gardenia. The top five differential metabolites were triethylamine, succinic acid, succinic acid semialdehyde, 2-phenylethanol, and o-xylene (Table 5).

Using the metabolites of Fr_7 as a reference (Figure 5f), the top three metabolites with the highest Z-scores in Fr_5 are 19(S)-HETE, 11,12-DiHETrE, and (4Z,7Z,10Z,13Z,16Z,19Z)-…d ethyl ester. The top three metabolites with the highest Z-scores in Fr_6 are l-cysteine, aureusidin, and estriol-16-glucuronide.

### 3.6. Pathways of Differential Metabolites in Samples

The KEGG pathway enrichment analysis was performed on the differential metabolites between each pair of samples. The top five differential metabolite pathways (Table 6) were biosynthesis of phenylpropanoids, biosynthesis of plant secondary metabolites, central carbon metabolism in cancer, protein digestion and absorption, and alanine, aspartate, and glutamate metabolism.

**Figure 6:**
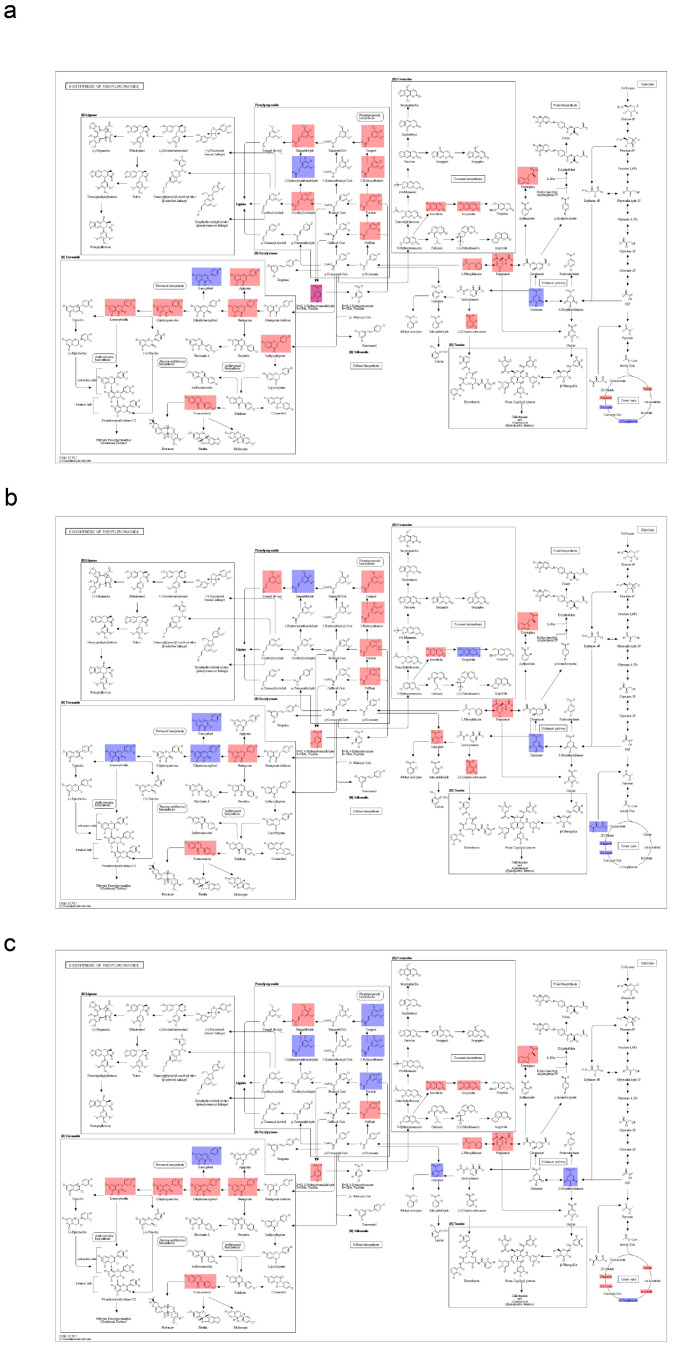
KEGG-enriched pathway map of amphetamine biosynthesis. (**a**): Fr_5 vs. Fr_6; (**b**): Fr_5 vs. Fr_7; (**c**): Fr_6 vs. Fr_7. Red indicates upregulation of the substance, while blue indicates downregulation of the substance. Boxes represent protein molecules, and circles represent metabolic molecules.

The phenylpropanoid metabolic pathway network for Fr_5, Fr_6, and Fr_7 is shown in (Figure 6). The types and concentrations of phenylpropanoid compounds synthesized showed significant differences among Fr_5, Fr_6, and Fr_7. The compounds in Fr_5 and Fr_6 were similar, while the differences between Fr_5 and Fr_7 were more pronounced.

## 4. Discussion

Variation constitutes the fundamental basis of biological evolution [14]. As a pivotal ornamental and developmental trait, the variation in petal number is likewise the outcome of the interplay between heredity and variation. From the perspective of evolutionary ecology, an increase in petal number acts as a ‘double-edged sword’: while it can potentially enhance reproductive success by expanding the floral display area and increasing attraction to pollinators, a complex floral structure alters resource allocation patterns, diverting energy originally intended for vegetative growth or other functions, and increases the risk of predation due to higher visibility [15]. Consequently, the variation and stability of this trait are collectively shaped by genetic bases, developmental programs, and environmental selection pressures.

Such phenotypic differentiation within a single species is primarily driven by two mechanisms: phenotypic plasticity arising from environmental heterogeneity in genetically identical individuals and the fixation of standing genetic variation within populations under natural selection [6]. In model plants, the molecular basis has been revealed to involve a multi-layered and precise regulatory network. For instance, specific transcription factors, such as PTL, indirectly regulate petal number by controlling the size of the boundaries between sepals [16], while CUC genes influence the physical space available for petal initiation by suppressing sepal growth [17]. Environmental signals, such as low temperature, can alter the developmental timing of floral buds, thereby creating possibilities for increasing petal count [18]. Regarding intrinsic regulation, hormonal pathways play a pivotal role; for example, gibberellins (GA) promote petal formation by degrading DELLA proteins to release transcription factors such as SPLs, which subsequently activate the expression of downstream MADS-box floral organ identity genes [19]. Collectively, these studies indicate that variation in petal number is the combined outcome of the dynamic interplay among the genetic blueprint, environmental signals, and endogenous hormonal networks, providing a theoretical framework for dissecting similar phenomena in non-model plants.

Within this framework, this study integrated transcriptomic and metabolomic analyses to systematically dissect the co-varying molecular networks underlying petal number variation in gardenia. The differential expression analysis identified a series of key transcription factors, predominantly enriched in the NAC, ERF, C2H2, MYB, and MADS-box families. Among them, MADS-box family members serve as core determinants of floral organ identity and developmental timing; the significant co-variation in their expression patterns with petal number strongly suggests that they play an evolutionarily conserved regulatory role in the establishment of floral whorls in gardenia [20,21,22]. Although genes from the NAC, ERF, and MYB families are widely known for their involvement in abiotic stress responses [23,24,25], their roles in developmental processes—such as floral meristem maintenance, organ boundary delineation, and hormone signal integration—have also been extensively documented. Therefore, the differential expression of these factors observed in this study is more likely associated with the intrinsic regulatory mechanisms governing the developmental trait of petal number itself.

Further analysis revealed that the expression levels of multiple ERF genes, including *GjERF28* and *GjERF39*, exhibited a precise gradient of co-variation with petal number. Notably, differentially expressed genes (DEGs) were significantly enriched in the ‘alanine, aspartate and glutamate metabolism’ pathway. Although conserved amino acid residues (e.g., alanine and aspartate) within specific ERF subfamilies share a nominal association with the pathway’s nomenclature [26], a functional linkage between these sequence motifs and metabolic pathway perturbations has not yet been established. In the absence of experimental evidence, this should be prudently interpreted as an intriguing correlative observation. Given that the ERF family functions as a central hub for integrating multiple hormonal signals, such as gibberellin and ethylene [24,27,28,29,30], we postulate that these ERF genes may influence the initiation patterns of organ primordia by modulating the hormonal microenvironment within the floral meristem; however, this hypothesis requires validation through functional experiments.

At the metabolic level, the phenylpropanoid biosynthesis pathway emerged as the primary site of differential accumulation. Furthermore, its known upstream regulators (e.g., MYB and bHLH factors) exhibited concurrent differential expression [31,32,33], thereby constructing a coherent landscape linking ‘transcriptional regulatory perturbations’ to ‘downstream metabolic phenotypes.’ However, it is crucial to clarify that the relationship observed between metabolic alterations and petal number represents a correlation. Underlying this observation, multiple possibilities exist: the metabolic shifts may represent secondary effects or physiological feedback resulting from alterations in biomass or carbon source allocation following changes in petal number; alternatively, they may constitute parallel outputs driven by the pleiotropic regulation of upstream core factors, such as ERF or MADS proteins. Based on the omics data and co-expression network analysis in this study, we hypothesize that ERF transcription factors likely mediate gibberellin biosynthesis or signaling, thereby coordinately regulating floral meristem activity and specific metabolic states to jointly determine the initial number of petal primordia. The key candidate genes identified herein provide crucial anchors for this hypothesis, although the precise causal relationships await final elucidation through subsequent experimental validation.

It should be acknowledged that a limitation of this study is the sampling at the full-bloom stage. Critical developmental decisions governing petal primordium initiation and organ number likely occur much earlier, during floral bud differentiation. Consequently, the transcriptomic and metabolomic profiles we captured primarily reflect the maintenance phase or the final steady state of the mature petals, rather than the initial decision-making events. We chose this stage for its practical advantages: it ensures unambiguous phenotypic scoring of petal number and enables synchronized sampling of tissues for integrated omics analyses. Nevertheless, the strong and consistent correlations we observed between petal number and the expression levels of key candidate genes (e.g., *GjERF28* and *GjMADS67*), even at this late stage, remain highly informative. These patterns suggest that these genes may play ongoing roles in maintaining organ identity, final size determination, or cellular homeostasis in petals, which are integral to the realization of the stable phenotype. Future studies employing high-resolution time-series sampling across early floral developmental stages will be essential to precisely delineate the temporal dynamics of these regulatory networks and to conclusively identify the upstream drivers of petal number variation in gardenia.

## 5. Conclusions

The corolla area, sepal number, and stamen number of gardenia flowers all increase with the number of whorled petals. A total of 18,720 genes are shared among the five-petal, six-petal, and seven-petal flowers. The gene expression level regulating the six-petal flower is higher than that of the five-petal and seven-petal flowers. The genetic and metabolic differences between the six-petal and five-petal gardenia are smaller, while the differences between the six-petal and seven-petal gardenia are larger. The commonalities among the three flower types are greater than their differences, and the differential genes are primarily located on chromosome 9. The differential genes between the six-petal flower and the five-petal and seven-petal flowers are primarily located extracellularly, with molecular functions mainly related to transmembrane transport and jasmonic acid responses. The differential genes between the five-petal and seven-petal flowers are mainly enriched in the cell wall, with molecular functions primarily involved in responses to oxygen-containing compounds. The differential genes in the five-petal, six-petal, and seven-petal gardenia flowers are all enriched in the biosynthesis of phenylpropanoids. The E-class genes of gardenia (*GjMADS79* and *GjMADS80*) are involved in various parts and stages of flower development. The expression patterns of the MADS-box and ERF gene family members in the five-petal, six-petal, and seven-petal gardenia flowers indicate that the upregulation of *GjERF28*, *GjERF39*, and *GjMADS67*, along with the downregulation of *GjMADS50*, *GjMADS59*, and *GjMADS60*, may influence the increase in the number of whorled petals in gardenia. There are 145 metabolites shared among the five-petal, six-petal, and seven-petal flowers. The differential metabolites primarily include triethylamine, succinic acid, succinaldehyde, 2-phenylethanol, and o-xylene. The pathways of the differential metabolites are mainly related to the biosynthesis of phenylpropanoids, biosynthesis of plant secondary metabolites, and metabolism of alanine, aspartate, and glutamate.

## Figures and Tables

**Table 1:** Statistics of the transcriptome sequencing. Q20, percentage of bases with base recognition accuracy higher than 99%; Q30, percentage of bases with base recognition accuracy higher than 99.9%.

Sample	Clean Reads/bp	Clean Reads/%	Clean Data/bp	Q20/%	Q30/%
Fr_5	42,844,284	93.36	6,426,642,600	98.01	94.54
Fr_6	40,703,316	93.37	6,105,497,400	98.07	95.12
Fr_7	37,395,350	93.28	5,609,302,500	98.24	94.62

**Table 2:** Statistics of sample comparison results.

Sample	Clean Reads	Total Mapped	Multiple Mapped	Uniquely Mapped	Map Events
Fr_5	42,844,284	39,699,613 (92.66%)	2,251,421 (5.67%)	37,448,192 (94.33%)	37,448,192
Fr_6	40,703,316	38,249,467 (93.97%)	1,958,780 (5.12%)	36,290,687 (94.88%)	36,290,687
Fr_7	37,395,350	35,155,842 (94.01%)	1,495,440 (4.25%)	33,660,402 (95.75%)	33,660,402

**Table 3:** GO enrichment analysis of the differentially expressed genes. —, indicates the absence of this GO Term.

Category	GO Term	Fr_5 vs. Fr_7	Fr_6 vs. Fr_5	Fr_6 vs. Fr_7
Cellular Component	Cell wall	5.35	6.31	3.71
	External encapsulating structure	5.35	6.31	3.71
	Extracellular region	4.51	—	—
	Cell junction	4.38	—	—
	Cell–cell junction	4.33	—	—
	Anchoring junction	4.33	—	—
	Plasmodesma	4.18	—	—
	Symplast	4.18	—	—
	Cell periphery	—	6.32	5.16
	Membrane	—	3.64	3.32
	Plasma membrane	—	—	2.93
Molecular Function	Glucose binding	5.33	3.81	—
	Inositol 3-alpha-galactosyltransferase activity	5.04	3.6	—
	Monosaccharide binding	5.04	3.6	—
	Glycogenin glucosyltransferase activity	4.03	—	—
	Glucosyltransferase activity	4.02	3.42	3.14
	UDP-glucosyltransferase activity	4.01	—	2.72
	Transmembrane transporter activity	—	4.98	
	Transporter activity	—	4.7	3.04
	(E,E)-geranyllinalool synthase activity	—	4.39	—
	Oxidoreductase activity, acting on paired donors, with incorporation or reduction in molecular oxygen	—	3.6	—
	4,8,12-trimethyltrideca-1,3,7,11-tetraene synthase activity	—	—	2.88
	(3E)-4,8-dimethyl-1,3,7-nonatriene synthase activity	—	—	2.88
Bioprocess	Response to oxygen-containing compound	5.25	—	—
	Response to stimulus	4.34	—	2.81
	Galactose metabolic process	4.1	—	—
	Response to iron ion	4.05	—	—
	Response to acid chemical	4.01	—	—
	Response to stress	3.99	—	—
	Transmembrane transport	—	6.18	—
	Secondary metabolite biosynthetic process	—	5.85	3.22
	Secondary metabolite process	—	5.19	3.16
	Anion transport	—	4.69	—
	Viral gene silencing in virus-induced gene silencing	—	4.48	—
	Anion transmembrane transport	—	4.15	—
	Transport	—	3.37	—
	Oxidation-reduction process	—	3.36	—
	Response to jasmonic acid	—	—	4.31
	Hormone metabolic process	—	—	3.22
	Regulation of hormone levels	—	—	3.16
	Negative regulation of circadian rhythm	—	—	2.93
	Molybdate ion export from vacuole	—	—	2.93
	Cellular response to inorganic substance	—	—	2.9
	Response to ethylene	—	—	2.74

**Table 4:** Differential transcription factors and their classification.

Treatment	Upregulated	Downregulated	Total	Transcription Factor Family
Fr_5 vs. Fr_7	80	87	167	AP2, ARF, ARR-B, B3, BBR-BPC, BES1, bHLH, bZIP, C2H2, C3H, CO-like, DBB, ERF, FAR1, G2-like, GATA, GeBP, GRAS, HB-other, HB-PHD, HD-ZIP, HSF, LBD, M-type_MADS, MYB, MYB_related, NAC, NF-YB, Nin-like, SBP, SRS, STAT, TCP, Trihelix, WRKY
Fr_6 vs. Fr_5	89	70	159	AP2, ARF, B3, BBR-BPC, BES1, bHLH, bZIP, C2H2, C3H, CO-like, DBB, ERF, FAR1, G2-like, GATA, GeBP, GRAS, HD-ZIP, HSF, LBD, MIKC_MADS, M-type_MADS, MYB, MYB_related, NAC, NF-X1, NF-YA, NF-YB, NF-YC, Nin-like, SRS, STAT, TALE, Trihelix, WRKY, YABBY, ZF-HD
Fr_6 vs. Fr_7	113	86	199	AP2, ARF, ARR-B, B3, BBR-BPC, BES1, bHLH, bZIP, C2H2, C3H, CO-like, DBB, Dof, ERF, FAR1, G2-like, GATA, GeBP, GRAS, HB-other, HD-ZIP, HSF, MIKC_MADS, M-type_MADS, MYB, MYB_related, NAC, NF-YA, NF-YC, Nin-like, RAV, SBP, STAT, TALE, TCP, Trihelix, VOZ, WRKY, YABBY, ZF-HD

**Table 5:** Top five differential metabolites. *m*/*z*: mass charge ratio; VIP: importance value; log_2_(FC): log_2_ value of the difference factor; *p* value: statistical *p*-value; KEGG number: KEGG compound ID.

Name	*m*/*z*	VIP	log_2_(FC)	*p* Value	Formula	KEGG Number
Triethylamine	101.06	1.30	0.28	2.18 × 10^−4^	C6H15N	C14691
Succinic acid	101.06	1.30	−1.11	3.35 × 10^−6^	C4H6O4	C00042
Succinic acid Semialdehyde	103.04	1.30	1.67	1.90 × 10^−3^	C4H6O3	C00232
2-Phenyl ethanol	105.07	1.30	0.41	2.94 × 10^−5^	C8H10O	C05853
o-xylene	107.09	1.30	2.35	5.53 × 10^−7^	C8H10	C07212

**Table 6:** Top five differential metabolite pathways. Hits: number of differential metabolites; FDR: post-correction false positive value; Impact: impact value. The larger the FDR value, the higher the possibility of false positives. The larger the impact value of metabolic pathways, the greater the impact of differential metabolites detected on the target pathway.

Pathway ID	Pathway Name	Total	*p* Value	Hits	FDR	Impact
map01061	Biosynthesis of phenylprop anoids	103	0	22	4.14 × 10^−6^	0.21
map01060	Biosynthesis of plant secondary metabolites	141	0	25	1.05 × 10^−5^	0.18
map05230	Central carbon metabolism in cancer	37	0	11	2.66 × 10^−4^	0.30
map04974	Protein digestion and absorption	47	0	12	3.97 × 10^−4^	0.26
map00250	Alanine, aspartate, and glutamate metabolism	28	0	9	6.23 × 10^−4^	0.32

## Data Availability

Data are available on request from the authors.
